# Evaluating the Mechanism of Cell Death in Melanoma Induced by the Cannabis Extract PHEC-66

**DOI:** 10.3390/cells13030268

**Published:** 2024-01-31

**Authors:** Ava Bachari, Nazim Nassar, Srinivasareddy Telukutla, Roby Zomer, Terrence J. Piva, Nitin Mantri

**Affiliations:** 1 The Pangenomics Lab, School of Science, RMIT University, Bundoora, VIC 3083, Australia or ava.bachari@gmail.com (A.B.); srinivasareddy.telukutla@rmit.edu.au (S.T.); 2 School of Health and Biomedical Sciences, RMIT University, Bundoora, VIC 3083, Australia; naz.nassar@rmit.edu.au (N.N.); terry.piva@rmit.edu.au (T.J.P.); 3 Faculty of Health, Charles Darwin University, Casuarina, NT 0810, Australia; 4 MGC Pharmaceuticals Limited, West Perth, WA 6005, Australia; roby@mgcpharma.co.uk; 5 The UWA Institute of Agriculture, The University of Western Australia, Perth, WA 6009, Australia

**Keywords:** melanoma, skin, cannabinoids, *C. sativa*, PHEC-66, apoptosis

## Abstract

Research suggests the potential of using cannabinoid-derived compounds to function as anticancer agents against melanoma cells. Our recent study highlighted the remarkable in vitro anticancer effects of PHEC-66, an extract from *Cannabis sativa*, on the MM418-C1, MM329, and MM96L melanoma cell lines. However, the complete molecular mechanism behind this action remains to be elucidated. This study aims to unravel how PHEC-66 brings about its antiproliferative impact on these cell lines, utilising diverse techniques such as real-time polymerase chain reaction (qPCR), assays to assess the inhibition of CB1 and CB2 receptors, measurement of reactive oxygen species (ROS), apoptosis assays, and fluorescence-activated cell sorting (FACS) for apoptosis and cell cycle analysis. The outcomes obtained from this study suggest that PHEC-66 triggers apoptosis in these melanoma cell lines by increasing the expression of pro-apoptotic markers (*BAX* mRNA) while concurrently reducing the expression of anti-apoptotic markers (*Bcl-2* mRNA). Additionally, PHEC-66 induces DNA fragmentation, halting cell progression at the G1 cell cycle checkpoint and substantially elevating intracellular ROS levels. These findings imply that PHEC-66 might have potential as an adjuvant therapy in the treatment of malignant melanoma. However, it is essential to conduct further preclinical investigations to delve deeper into its potential and efficacy.

## 1. Introduction

Melanomas are cancerous cells arising from epidermal melanocytes [1]. While melanoma accounts for ~6% of skin cancers, it is the cause of >80% of related skin cancer deaths [2]. This cancer is highly prone to metastasising and can generate tumours through local and distant invasions [3] and has been shown to be highly resistant to traditional treatments [4]. Melanoma development is influenced by various factors, including chronic inflammatory conditions like psoriasis and eczema, genetic factors, and prolonged exposure to environmental hazards such as ultraviolet (UV) radiation [5].

The current pharmacological treatment options for metastatic melanoma are limited, resulting in a low median survival time of ~8–9 months, with <15% of patients surviving beyond three years [6]. Approximately 50% of melanomas have BRAF mutations, with the majority being BRAF^V600E^, which causes intrinsic activation of the BRAF-MEK-ERK-MAPK signalling pathway, also known as the Mitogen-activated protein kinase (MAPK) pathway [7,8], leading to enhanced melanoma cell proliferation and survival [7]. Inhibition of the MAPK signalling pathway through agents such as dabrafenib, vemurafenib, binimetinib, encorafernib, trametinib, selumetinib, and cobimetinib, which bind to signal cascade kinases BRAF and MEK, has become a crucial treatment strategy for patients with BRAF-mutant melanoma [9,10,11,12].

Immunotherapy has revolutionised cancer treatment in recent years. Tumour necrosis factor-alpha (TNFα), interleukin-2 (IL-2), and interferon-gamma (IFNγ) are shown to be critical in fighting melanoma cells [13,14]. Moreover, immune checkpoint inhibitors (ICIs) (e.g., pembrolizumab, nivolumab, atezolizumab, ipilimumab, and relatlimab) have demonstrated effective antitumour activity in melanoma. ICIs, including PD-1 and its ligand PD-L1, along with CTLA-4, are evolving treatment options for several types of cancer [15,16,17,18,19]; however, a limited number of patients have been shown to benefit from such therapy [20,21]. Combined therapy between BRAK/MEK and immunotherapy or “checkpoints” effectors has enhanced melanoma patient survival [22]. However, sequential administration instead of concurrent treatment is recommended to avoid treatment-limiting toxicities [22]. Considering the above-mentioned issues, an additional treatment strategy is imperative to mitigate the adverse effects of the current pharmacotherapies and complement the single-drug treatment approach.

Evidence suggests that certain plant-derived natural compounds, including polyphenols, flavonoids, and terpenes, may possess anticancer properties helpful in treating melanoma [23]. For instance, some compounds present in cannabis may exert antitumour effects and potentially impede the growth and spread of melanoma cells [24]. The endogenous cannabinoid system (ECS) is a ubiquitous neuromodulator system that modulates a broad range of physiological functions in the human body [25]. This system comprises cannabinoid receptors and their endogenous ligands, as well as the enzymes that synthesise and degrade them [25]. The primary mediators of the ECS system are the cannabinoid receptors CB1 and CB2, which are distributed widely in the central nervous and periphery system [26]. These G protein-coupled receptors are known to influence various intracellular signalling pathways that regulate different molecular processes, such as gene transcription, cell motility, angiogenesis, and apoptosis [26,27]. Cannabinoids can also ligand via the G protein-coupled receptor GPR55 and the peroxisome proliferator-activated receptors (PPARs) [28]. Moreover, recent research has revealed that cannabinoids can influence specific transient receptor potential (TRP) channels [29]. There are three subfamilies of TRP channels: TRP vanilloid (TRPV), TRP ankyrin (TRPA), and TRP melastatin (TRPM) [29]. Notably, six TRP channels have been identified as mediators of cannabinoid activity: TRPV1, TRPV2, TRPV3, TRPV4, TRPA1, and TRPM8 [29].

Previous studies have demonstrated that the activation of CB1 and CB2 by external plant-derived compounds results in the inhibition of early tumour growth in various malignancies, including non-small cell lung carcinoma [30], prostate cancer [31], and melanoma [32], where these receptors function as mediators that stimulate diverse molecular signalling pathways [26]. Recently, we demonstrated that the *Cannabis sativa* (*C. sativa*)-derived extract PHEC-66 inhibited the growth and motility of different melanoma cells [33]. In this study, we have explored the mechanisms underlying the effects of PHEC-66 on melanoma cells, which may reveal potential targets for drug development and shed light on the impact of cannabinoids on these cells.

## 2. Materials and Methods

### 2.1. Materials

RPMI-1640 media, heat-inactivated foetal bovine serum (FBS), streptomycin, penicillin, PowerUp SYBR Green Master Mix, Annexin V-FITC (11-8005-74), propidium iodide (PI) staining in annexin binding buffer (00-6990-42), Thiazoyl blue tetrazolium bromide (MTT), and 5-(and-6)-carboxy-2′,7′-dichlorodihydrofluorescein (DCF-DA) were obtained from Thermo Fisher Scientific (Melbourne, Australia). AM251 (A6226), AM630 (SML0327), and TRIzol reagent were purchased from Sigma (Sydney, Australia). The Propidium Iodide flow cytometry kit (Abcam139418) was purchased from Abcam (Melbourne, Australia). The SensiFAST^TM^ cDNA Synthesis Kit was obtained from Meridian Bioscience (Cincinnati, OH, USA), and the PCR primers were purchased from Bioneer (Melbourne, Australia).

#### Cell Culture

Human melanoma MM418-C1 (primary (1°) melanoma possessing the oncogenic BRAF^V600E^ mutation), MM329 (Primary (1°) melanoma possessing wild type BRAF (BRAF^WT^)), and MM96L (metastatic or secondary (2°) melanoma possessing the oncogenic BRAF^V600E^ mutation) cells, and human immortalised keratinocytes (HaCaT), were used in this study. The MM418-C1, MM329, MM96L, and HaCaT cells were kindly supplied by Prof Nicholas Hayward and Peter Parsons of the Queensland Institute of Medical Research, Brisbane, Australia.

### 2.2. Methods

Cells were cultured in RPMI-1640 tissue culture medium supplemented with 10% (*v*/*v*) FBS and 1% (*v*/*v*) penicillin and streptomycin. The cells were incubated at 37 °C in a humidified 5% CO_2_ incubator and passaged every 3–4 days until they reached 80–90% confluency.

#### 2.2.1. Quantitative Polymerase Chain Reaction (qPCR)

In 6-well plates, cells were seeded at a density of 5 × 10^5^ cells/well in RPMI media supplemented with 10% (*v*/*v*) FBS and 1% (*v*/*v*) penicillin and streptomycin. The cells were then incubated in a humidified 5% CO_2_ incubator at 37 °C until they reached ~75% confluency. The cells were then treated with RPMI media containing PHEC-66-IC_50_ (half-maximal inhibitory concentration of PHEC-66) for 24 h. Total RNA was extracted using TRIzol™ Reagent according to the manufacturer’s instructions. The RNA was quantified at 260 nm using a spectrophotometer (Nanodrop ND1000), and the purity was assessed by measuring the absorbance ratios at 260/280 nm and 260/230 nm. SensiFAST^TM^ cDNA Synthesis Kit was used to synthesise cDNA from 1 µg of total RNA. Each transcript quantification was performed in triplicate. Then, 1 µL of cDNA was mixed with 3.6 µL of PowerUp SYBR Green Master Mix, 0.4 µL of each forward and reverse primer, and 2.9 µL of DEPC H2O. The QuantStudio^TM^ 7 Real-Time PCR (Thermofisher, Melbourne, Australia) was used to perform qPCR with the following thermal cycling conditions: an initial activation at 95 °C for 2 min, 39 cycles of 95 °C for 5 s, 60 °C for 10 s, 72 °C for 15 s, and a melting curve from 65 °C to 95 °C. The 2^–ΔΔCq^ method was used for relative quantitative analysis of the expression of the genes of interest normalised to the housekeeping gene glucuronidase (GUSB). The expression was calculated as n-fold induction of the gene of interest in treated cells relative to that of untreated control cells, which was expressed as unity (1).

#### 2.2.2. CB1/CB2 Receptors Antagonism Assay

The cytotoxicity of antagonists combined with PHEC-66 on MM418-C1, MM329, and MM96L cells was evaluated using the MTT assay. Cells were seeded into 96-well plates at a density of 3000–10,000 cells per well depending on their doubling times and allowed to adhere for 24 h at 37 °C and 5% CO_2_. After 24 h incubation, this medium was replaced with tissue culture medium containing CB1 and CB_2_ antagonists combined with PHEC-66 dissolved in (0.05% *v*/*v*) DMSO. DMSO was used as the solvent control. After 48 h incubation, the medium containing the test compounds was aspirated, and 100 µL of culture media containing 5 mg/mL MTT was added to each well. The cells were incubated for 3 h in the dark at 37 °C, after which this solution was removed, and 100 µL DMSO was added to each well to solubilise the crystallised formazan product. The plates were read on a microplate reader at 570 nm and a reference wavelength of 630 nm. The growth percentage of inhibition was calculated as 100 − [(Mean OD of the treated cell × 100)/Mean OD of vehicle-treated cells (DMSO)]. The IC_50_ values were calculated using Prism Software. All experiments were performed three times independently.

#### 2.2.3. Reactive Oxygen Species (ROS)

The cells were plated in a 6-well plate with a density of 4 × 10^5^ cells per well and allowed to adhere overnight. The cells were then treated with PHEC-66 at a concentration of 50% and 100% of their respective IC_50_ values for 24 h. After treatment, the cells were collected and suspended in 1 mL of phosphate-buffered saline (PBS) containing 10 μM DCF-DA at room temperature (RT) and placed in the dark for 15 min. Next, the cells were washed with PBS to remove excess dye before being examined using a BD LSRFortessa Cell Analyzer.

#### 2.2.4. Cell Cycle Arrest Assay

The cells were plated in a 6-well plate at a density of 4 × 10^5^ cells per well and allowed to adhere overnight. The cells were treated with PHEC-66 at a concentration of 50%, 100%, and 200% of their respective IC_50_ values for 24 h. The cells were trypsined, rinsed with ice-cold PBS, and then fixed with 70% ice-cold ethanol. Next, the cells were washed with PBS and stained with propidium iodide (PI) staining buffer. Finally, 10,000 cells from each sample were examined for propidium iodide fluorescence using the BD LSRFortessa^TM^ Cell Analyzer Flow Cytometer.

#### 2.2.5. Annexin V-FITC/PI Staining Assay

To determine the apoptotic events induced by PHEC-66, annexin V and PI staining were used, following the manufacturer’s protocol for the Dead Cell Apoptosis Kit with annexin V-FITC and PI and analysed using flow cytometry. The cells were plated in a 6-well plate at a density of 4 × 10^5^ cells per well and allowed to adhere overnight. The cells were treated with PHEC-66 at a concentration of 50%, 100%, and 200% of their respective IC_50_ values for 48 h. The cells were harvested, washed with PBS, and resuspended in 100 μL annexin V binding buffer as per the manufacturer’s instructions. Next, Annexin V (5 μL) and PI (1 μL of 100 μg/mL) were added to the suspended cells and the cells incubated for 15 min at RT in the dark. Next, the cells were washed with PBS to remove excess dye before being examined. Subsequently, a total of 10,000 events were acquired to assess green fluorescence using a BD LSRFortessa Cell Analyzer. Cells were considered to be apoptotic if they were Annexin V^+^/PI^−^ (early apoptotic) or Annexin V^+^/PI^+^ (late apoptotic/necrotic). Live cells were defined as Annexin V^−^/PI^−^, and cell debris was Annexin V^−^/PI^+^ as necrotic.

### 2.3. Statistical Analysis

Statistical analyses were conducted using GraphPad Prism version 8 (GraphPad Software Inc., San Diego, CA, USA). Bar plots and graphs were generated using GraphPad software. The data are presented as mean ± standard deviation (SD) for three independent experiments. For comparisons involving three or more groups, either one-way or two-way ANOVA was performed, followed by Tukey’s multiple comparison test. In cases where there were only two groups, the Mann–Whitney U test was used. A two-sided *p*-value of less than 0.05 was considered statistically significant, denoted as * (*p* < 0.05), ** (*p* < 0.01), *** (*p* < 0.001), or **** (*p* < 0.0001).

## 3. Results

We have previously reported that PHEC-66 reduced the viability of human melanoma cell lines (MM418-C1, MM329, and MM96L) in a concentration-dependent manner [33]. PHEC-66 decreased, to a lesser extent, the viability of non-transformed epidermal cells [33]. In the melanoma cells, the IC_50_ for PHEC-66 was approximately half of that seen in the untransformed HaCaT cells, as seen in Table 1. Inspired by the remarkable melanoma cancer cell growth inhibition properties of PHEC66, in this study, we have investigated the mechanism of cell growth inhibition.

### 3.1. Quantitative Polymerase Chain Reaction (qPCR)

**Figure 1:**
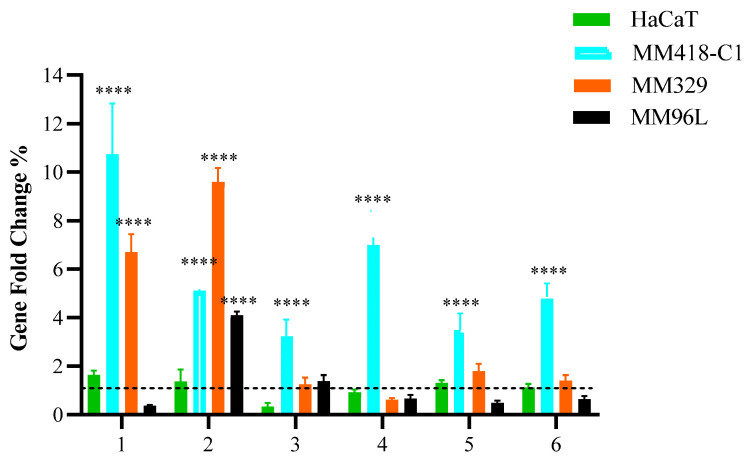
Effect of PHEC-66 on the expression of cell membrane receptors. Melanoma (MM418-C1, MM329, and MM96L) and keratinocyte-derived cells (HaCaT) were treated with PHEC-66 at its IC_50_ concentration for 24 h. Asterisks indicate statistically significant differences between PHEC-66-IC_50_-treated melanoma cell lines and the corresponding non-treated cells. All data represent the mean ± SEM of three independent experiments (**** *p* < 0.0001). The dotted line represents untreated cells which ranked number one across all cells on the *Y*-axis scale.

To comprehend the effect of PHEC-66, we examined its effect on the expression of membrane cannabinoid receptors, including CB1, CB2, TRPV1, TRPV2, TRPM8, and GPR55, as seen in Figure 1.

Following PHEC-66-IC_50_ treatment of MM418-C1 cells, there was a considerable increase in the expression of some of the receptor genes compared to the untreated cells. When the MM418-C1 cells were treated with PHEC-66-IC_50_, there was a significantly higher expression of CB1, CB2, TRPV1, TRPV2, TRPM8, and GPR55 genes, while in the MM329 cells, only CB1 and CB2 significantly increased, and there were insignificant changes in the expression of TRPV1, TRPV2, TRPM8, and GPR55. Additionally, PHEC-66 at its IC_50_ concentration significantly induced the expression of CB2 genes in MM96L and decreased the expression of CB1, TRPV2, TRPM8, and GPR55 genes but insignificantly. HaCaT cells did not exhibit significant changes in the expression of these genes after treatment with PHEC-66-IC_50_. These findings suggest that CB1 and CB2 play a significant role in the growth-inhibitory mechanism of PHEC-66 in melanoma cell lines. Consequently, the impact of CB1 and CB2 was further investigated by employing antagonists to block their activity and examine the effects this inhibition had on the viability of melanoma cells.

### 3.2. CB1 & CB2 Antagonist

**Figure 2:**
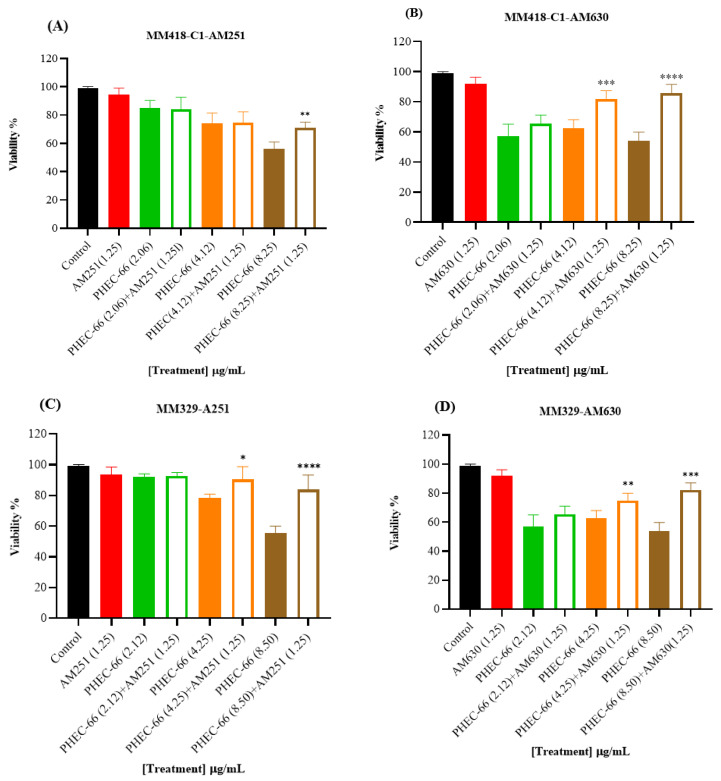

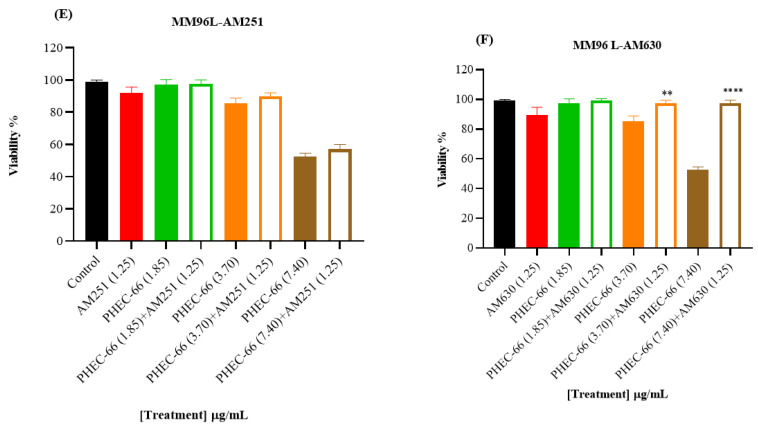
The effect of PHEC-66 extract on the viability of melanoma cells treated with CB1 and CB2 antagonists. MM418-C1 cells treated with PHEC-66 in the presence or absence of (**A**) CB1 antagonist (1.25 µg/mL AM251), (**B**) CB2 antagonist (1.25 µg/mL AM630). MM329 cells treated with PHEC-66 in the presence or absence of (**C**) CB1 antagonist (1.25 µg/mL AM251), (**D**) CB2 antagonist (1.25 µg/mL AM630). MM96L cells treated with PHEC-66 in the presence or absence of (**E**) CB1 antagonist (1.25 µg/mL AM251), (**F**) CB2 antagonist (1.25 µg/mL AM630). The data are presented as means ± standard deviations for each group (*n* = 3). Significant differences between groups are indicated by * *p* < 0.05, ** *p* < 0.01, *** *p* < 0.001, **** *p* < 0.0001.

In order to confirm the qPCR results, the melanoma cells were exposed to CB1 (AM261) and CB2 (AM630) antagonists to block these receptors. This approach aimed to confirm whether blocking either the CB1 or CB2 receptor would influence the impact of PHEC-66 on cell viability. The results reveal a significant increase in cell viability for the MM418-C1, MM329, and MM96L cell lines when treated with CB2 antagonist (AM630) plus PHEC-66 compared with untreated cells. Similarly, administering CB1 antagonist (AM251) plus PHEC-66 to these melanoma cell lines significantly improved treated MM418-C1 and MM329 cell viability compared with the untreated cells (Figure 2).

When the melanoma cells were exposed to PHEC-66 alone, their viability was approximately 55%. The addition of PHEC-66 to AM251-treated cells increased cell viability to 71% for MM418-C1 cells and 83% for MM329 cells (Figure 2A,C). However, for MM96L cells, using the CB1 antagonist to block PHEC-66 did not change the viability of these cells compared to untreated cells (Figure 2E).

### 3.3. Reactive Oxygen Species (ROS)

**Figure 3:**
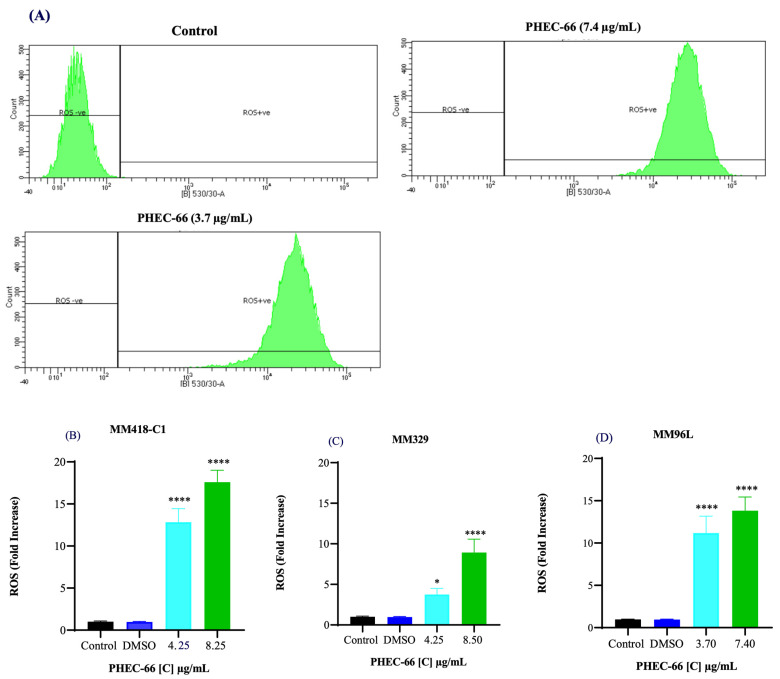
Effect of PHEC-66 treatment on cellular ROS level in melanoma cells. (**A**) ROS levels for MM96L were measured using BD LSRFortessa, (**B**) MM418-C1, (**C**) MM329, and (**D**) MM96L were treated for 24 h with PHEC-66 h at both 50% and 100% of its IC_50_ concentration. Data are expressed as the means ± standard deviations of triplicate experiments (*n* = 3). Significant differences between PHEC-66-treated cells and vehicle-control cells are marked with * *p* < 0.05, **** *p* < 0.0001.

We investigated whether PHEC-66 increased ROS levels in these cells. We exposed the melanoma cells to two different PHEC-66 concentrations (50% and 100% of their corresponding IC_50_ values) for 24 h. PHEC-66 at 50% of its IC_50_ concentration caused a 12-, 3-, and 11-fold increase in ROS levels in MM418-C1, MM329, and MM96L cells, respectively, compared to the corresponding untreated controls. The ROS levels increased by factors of 17, 8, and 13 when PHEC-66 was introduced to these cells at its IC_50_ concentration, and this augmentation was not attributable to the solvent carrier, DMSO (Figure 3).

### 3.4. Effect of PHEC-66 on Cell Cycle Dynamics

**Figure 4:**
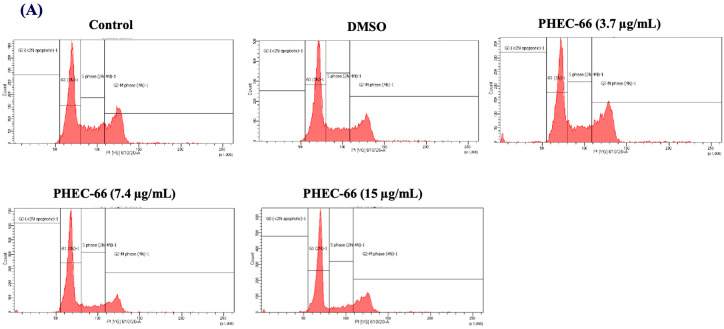

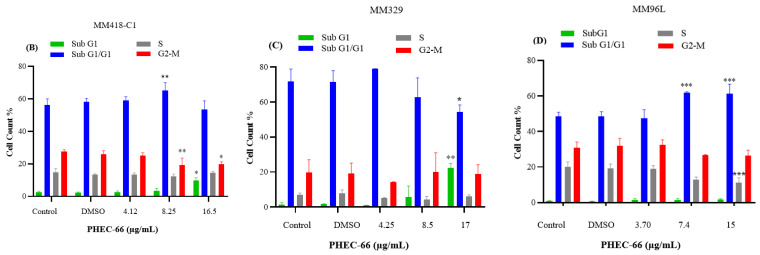
The effect of PHEC-66 treatment on melanoma cell cycle kinetics was assessed for three different cell lines. These cells were exposed to PHEC-66 at concentrations equivalent to 50%, 100%, and 200% of their respective IC_50_ values, and the treatment duration was 24 h. (**A**) Cell cycle distribution for MM96L was analysed for MM96L using BD LSRFortessa, (**B**) MM418C1, (**C**) MM329, and (**D**) MM96L. The data presented represent the mean ± standard deviations for each group (*n* = 3). Significant differences between groups are indicated * *p* < 0.05, ** *p* < 0.01, *** *p* < 0.001.

In order to further comprehend the mechanism by which PHEC-66 inhibits the growth of cancer cells, a cell cycle analysis of its effect on melanoma cells was investigated. PHEC-66 (16.5 µg/mL) treatment for 24 h increased the percentage of MM418-C1 cells in subG1, indicating damaged and fragmented DNA, which was not observed in the untreated controls (Figure 4A). A similar observation was made for MM329 cells, when PHEC-66 (17 µg/mL) increased the percentage of cells in subG1 (20%) compared to the untreated controls (1.5%). This increase in the number of cells in the sub-G1phase of MM329 cells corresponded to a reduction in the number of cells in the subG1/G1 phase (Figure 4B). Of interest was that in MM96L cells, the PHEC-66 (15 µg/mL) treatment increased the percentage of cells in the subG1/G1 phase by ~13%, which corresponded to the reduction (~10%) in cells in the S phase (Figure 4C).

### 3.5. Apoptosis Assay

**Figure 5:**
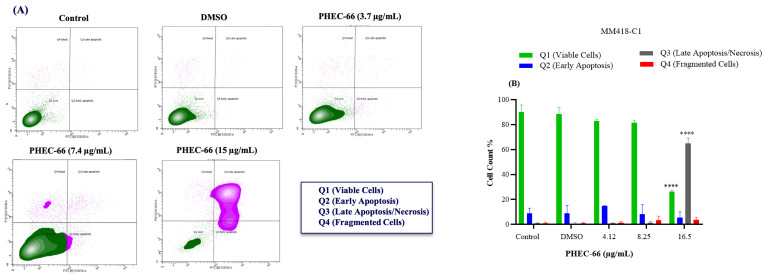

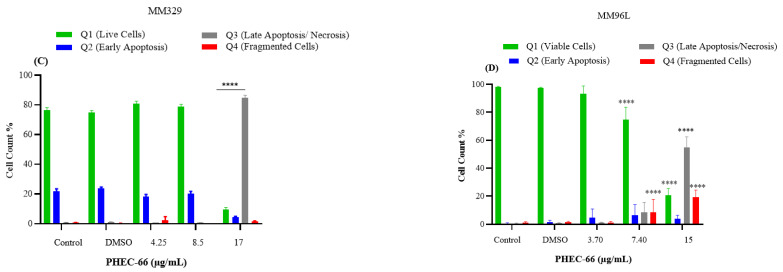
The effect of PHEC-66 treatment on melanoma was assessed for three different cell lines. These cells were exposed to PHEC-66 at concentrations equivalent to 50%, 100%, and 200% of their respective IC_50_ values, and the treatment duration was 48 h. (**A**) Apoptosis for MM96L was analysed using BD LSRFortessa, (**B**) MM418-C1, (**C**) MM329, and (**D**) MM96L. Data are expressed as means ± standard deviations of each group (*n* = 3). Significant differences between groups are marked with **** *p* < 0.0001.

After subjecting MM418-C1 cells to PHEC-66 treatment at a concentration of 17 µg/mL for 48 h, the population of viable cells decreased by 64%, due to their progress to a late apoptosis/necrosis state (Figure 5A). In MM329 cells, using 17 µg/mL of PHEC-66 treatment increases the number of cells experiencing late apoptosis (from 1% to 84%) (Figure 5B). Concerning the third cell line, MM96L, Figure 5C displays an increase in late apoptosis by ~64% following the application of 15 µg/mL of PHEC-66.

### 3.6. BAX and Bcl-2 Gene Expression

In order to gain a more comprehensive understanding of the underlying mechanism of PHEC-66 in triggering apoptosis, we assessed its effect on the expression of *Bcl-2*, an anti-apoptotic protein, and *BAX*, a pro-apoptotic protein. The administration of PHEC-66 led to a reduction in the expression of the anti-apoptotic *Bcl-2* gene while increasing the expression of the pro-apoptotic *BAX* gene when compared to untreated cells. This reaffirms the previous findings that PHEC-66 induces apoptosis in melanoma cell lines.

PHEC-66 reduced the release of *Bcl-2* in MM418-C1 and MM329, leading to augmented expression of *BAX* in these cell lines; nevertheless, no significant changes in the expression of *BAX* and *Bcl-2* were observed in MM96L. In MM418-C1 cells treated with PHEC-66-IC_50_, the expression of *Bcl-2* gene decreased significantly, while *BAX* gene expression notably increased five-fold compared to untreated cells. In MM329 cells, there was approximately a 1.9-fold increase in *BAX* gene expression, as indicated in Table 2.

Interestingly, when we applied PHEC-66-IC_50_ to non-cancerous keratinocyte cells, there was a 2.27-fold increase in the expression of *Bcl-2* and no reduction in *BAX* expression. This suggests that PHEC-66 is less likely to induce apoptosis (cell death) in non-cancerous cells than in melanoma cells, supporting our earlier findings regarding cell death.

## 4. Discussion

We have previously shown that PHEC-66 exerts a cytotoxic effect on several melanoma cell lines [33]. PHEC-66 prevented the formation of cell colonies, impeded cell migration, induced cellular immobility, and induced morphological changes indicative of apoptosis [33].

Using the quantitative polymerase chain reaction (qPCR), we observed PHEC-66 at its IC_50_ concentration induced differential changes in the expression of receptor genes of the melanoma cell lines. These results suggest that the signalling pathways triggered by PHEC-66 may involve CB1 and CB2 receptors. Moreover, it is evident that the response to PHEC-66 treatment differs among various cell lines, with MM418-C1 cells being the most responsive in terms of gene expression changes as indicated in Table 3. Drozd et al. observed the anticancer effect of cannabinoids by activating CB1 and CB2 receptors in tissue-cultured Lewis lung adenocarcinoma cells [34]. Another preclinical study, both in vivo and in vitro, also demonstrated that cannabinoids exert their anticancer effects primarily by inducing apoptosis through CB1, CB2, and TRPV1 receptors [35]. Furthermore, cannabinoids have been shown to decrease the expression of the epidermal growth factor receptor (EGFR) as well as plasminogen activator inhibitor-1 (PAI-1) while increasing the expression of the tissue inhibitors of matrix metalloproteinases-1 (TIMP-1) in non-small cell lung cancer (NSCLC) A549, H358, and H460 cell lines, and human-derived NSCLC cells via CB1, CB2, and TRPV1 [36]. Therefore, in a broad sense, cannabis extract, such as PHEC-66, which is primarily composed of CBD (60%), hinders cell proliferation [36], diminishes cell migration, and suppresses the invasive potential of melanoma cells [37], thereby inhibiting angiogenesis and impeding the development of metastases [36].

Nonetheless, fully establishing the effect of PHEC-66 on CB1/CB2 receptors in melanoma cells necessitates further investigations at both the protein level and functional aspects. These additional studies should explore whether the increased receptor expression results in enhanced receptor functionality and evaluate the potential therapeutic implications of modulating these receptors in melanoma. This comprehensive analysis would provide a deeper understanding of the mechanisms underlying PHEC-66′s impact on melanoma and its potential for future therapeutic development. Consequently, the impact of CB1 and CB2 was further investigated by employing antagonists to block their activity and examine the effects this inhibition had on the viability of melanoma cells as seen in Table 3.

The interaction between PHEC-66 and its receptors was examined in the presence of specific antagonists, namely, AM251 and AM630, which selectively block CB1 and CB2 receptors, respectively. The use of these specific CB1 and CB2 receptor blockers confirmed that the reduction in growth caused by PHEC-66 was primarily associated with CB2 receptors in all examined melanoma cells.

These findings suggest that the CB1 receptor may be involved to a minor extent in PHEC-66 activity on MM96L cells, while CB2 antagonist significantly blocked PHEC-66 cytotoxicity in all tested cell lines. These results aligned with a previous study that demonstrated the cytotoxic impact of cannabis sativa extract on bladder urothelial carcinoma cell lines, including T24 and HBT-9 cell lines. Notably, the cytotoxic effect was diminished when CB1 and CB2 antagonists were introduced [38].

Cannabinoids have been shown to induce ER stress, resulting in increased ROS levels, which can trigger apoptosis in non-melanoma cells [39,40,41]. The administration of PHEC-66 at half of its IC_50_ dose resulted in a significant increase in ROS levels within melanoma cells. Moreover, a more substantial elevation in ROS levels was observed when PHEC-66 was applied at its full IC_50_ dose.

ROS is known to be important for CBD-induced cell death in glioma and leukaemia cells [42,43]. Reduced mitochondrial function leads to higher production of ROS through a process known as electron leakage in the electron transport chain of mitochondria [44]. Moreover, recent studies suggest elevated ROS levels are associated with initiating apoptosis and autophagy [37].

Multiple studies have consistently demonstrated the involvement of endocannabinoids and cannabinoids in exerting antiproliferative effects through metabolic pathways, particularly those involving ROS [37,41,42,43,44]. Park et al. observed a substantial elevation in ROS levels when treating head and neck squamous cell carcinoma (HNSCC) lines with the endocannabinoid anandamide (AEA) [45]. Utilising such cell lines, increased ROS production was observed after AEA treatment. Moreover, antioxidants such as hydrogen peroxide (H_2_O_2_) partially reversed the AEA-dependent inhibition of cell proliferation by inducing oxidative stress and damaging cellular components such as DNA, proteins, and lipids [45]. This oxidative damage triggers cell cycle arrest and programmed cell death (apoptosis), ultimately preventing cells from dividing and proliferating [46]. AEA treatment increased intracellular ROS levels in mice cholangiocytes [47]. When these cells were treated with 10 µM AEA for 2 h, ROS levels rose 30-fold [47]. These findings imply that cannabinoids, such as AEA, can trigger ROS generation and accumulation in different cell types. Consequently, this process can induce alterations in cellular signalling pathways, potentially prompting apoptosis cell death. However, further investigation is required to understand the precise mechanism by which PHEC-66 exerts its action on these melanoma cells.

Apoptotic cell death is generally associated with alterations in the cell cycle program [48,49]. When MM418-C1 cells were exposed to 16.5 µg/mL PHEC-66, there was an increase in the sub- G1 phase, which typically indicates cells with fragmented or degraded DNA, a characteristic feature of apoptosis/necrosis [49]. PHEC-66 treatment of MM329 resulted in an elevated sub- G1 phase (indicating apoptotic/necrotic cells) and a corresponding decrease in cells in the sub- G1/G1 phase [48]. In the MM96L cells, there was a notable increase in cells in the sub- G1/G1 phase and a significant reduction in the S phase after PHEC-66 administration as seen in Table 3. DNA synthesis occurs in the S phase, so this decrease suggests that the G1/S cell cycle checkpoint has been blocked [50]. In light of this, the impact of PHEC-66 treatment on three different cell lines highlights its impact on their cell cycle phases. It suggests that PHEC-66 treatment may induce apoptosis and affect cell distribution across various cell cycle phases in these cell lines. These findings are essential for understanding the mechanism of action of PHEC-66 on these cells, and further studies are needed on PHEC-66 to understand why it affected these cells differently.

In order to gain a more comprehensive understanding of the underlying mechanism of PHEC-66 in triggering apoptosis, we assessed its effect on the expression of *Bcl-2*, an anti-apoptotic protein, and *BAX*, a pro-apoptotic protein.

*BAX* and *Bcl-2* are both members of the *Bcl-2* family, *BAX* is a central regulator in the intrinsic apoptosis pathway [51]. When stimulated by apoptotic signals, it becomes activated and assembles into oligomers on the mitochondrial outer membrane (MOM), promoting its permeabilization and releasing cytochrome c during mitochondrial-mediated intrinsic apoptosis [52,53], whereas *Bcl-2* acts as an inhibitor of apoptosis by forming a heterodimer with *BAX*, ensuring the inhibition of *BAX* activity, leading to cell survival sustained by regulating the Ca^2+^ concentration [54]. The balance between *Bcl-2* and *BAX* proteins is critical in determining whether cytochrome c is released from the mitochondria and whether apoptosis is initiated [55]. High levels of *Bcl-2* tend to inhibit cytochrome c release and promote cell survival, while increased *BAX* levels favour cytochrome c release and apoptosis. Hence, the ratio of *Bcl-2* to *BAX* can be a critical regulatory factor in determining a cell’s fate regarding apoptosis. The administration of PHEC-66 led to a reduction in the expression of the anti-apoptotic *Bcl-2* gene while increasing the expression of the pro-apoptotic *BAX* gene when compared to untreated cells. This reaffirms the previous findings that PHEC-66 induces apoptosis in melanoma cell lines. The contrasting cellular dynamics observed in HaCaT cells, when compared to melanoma cell lines, offer valuable context for interpreting the impact of PHEC-66. Specifically, the observed rise in *Bcl-2* expression suggests a pro-survival effect, as it appears to coincide with a decrease in *BAX* expression, resulting in a favourable ratio that suppresses pro-apoptotic signals. This dual effect implies a cellular response that leans towards enhanced cell viability in the HaCaT cell line. Such observations underscore the importance of recognising cell type-specific responses to PHEC-66 and suggest that PHEC-66 may have a relatively reduced inhibitory effect on HaCaT cells, in contrast to its potential impact on melanoma cell lines. Further investigations are warranted to elucidate the underlying molecular mechanisms governing these effects and to ascertain the broader implications for the application of PHEC-66 in the context of cell-specific therapeutic strategies.

## 5. Conclusions

In conclusion, we have shown that PHEC-66 impedes the growth of MM418-C1, MM329, and MM96L melanoma cells. This inhibitory effect arises from interactions with CB1 and CB2 receptors. PHEC-66’s impact extends to the modulation of cell cycle progression, particularly evident in the sub G1 and sub G1/G1 phases. Furthermore, PHEC-66 influences metabolic pathways by inducing the accumulation of ROS within these cells, thereby tilting the balance toward pro-apoptotic signalling pathways while diminishing anti-apoptotic ones. All these actions together start the process of apoptosis and slow down the growth of melanoma cells. Further studies are required for a comprehensive understanding of its potential use in advanced-stage melanoma treatment, preferably involving more sophisticated models and assessing its viability within combination therapies.

## Figures and Tables

**Table 1:** Comparison of the IC_50_ value for epidermal cells treated with PHEC-66. Data represent the mean ± standard deviation of three independent experiments performed in triplicate [33].

Cell Lines	IC_50_ (μg/mL)
HaCaT	13.37 ± 1.90
MM418-C1	8.21 ± 0.75
MM329	8.47 ± 0.14
MM96L	7.41 ± 0.94

**Table 2:** Evaluation of the gene expression level of *BAX* and *Bcl-2* in melanoma cells (MM418, MM329, MM96L) and HaCaT cells following treatment with IC_50_ of PHEC-66 for 24 h in comparison to untreated cells.

Cell Lines	*BAX*	*Bcl-2*	Ratio of *BAX/Bcl-2*
HaCaT	0.62	2.27	0.27
MM418-C1	5.25	0.84	6.25
MM329	1.90	0.79	2.40
MM96L	0.90	0.61	1.47

**Table 3:** Effect of PHEC-66 on various mechanisms of melanoma cell lines. PHEC-66 either increased (↑), had no effect (=) or reduced (↓) gene expression and ROS levels, while the CB antagonists either reduced (+) or had no effect (−) on PHEC-66’s cytotoxic effect.

Effect	MM418-C1	MM329	MM96L
CB1 Expression CB2 Expression	↑ ↑	↑ ↑	↓ ↑
CB1 Antagonist CB2 Antagonist	+ +	+ +	− +
Cell Cycle Arrest	Sub G1 Damage and fragmented DNA	Sub G1 Damage and fragmented DNA	Sub G1-G1
ROS Level	↑	↑	↑
Apoptosis Effect	Apoptosis	Apoptosis	Apoptosis/Necrosis
*BAX* Expression *Bcl-2* Expression	↑ ↓	↑ ↓	= =

↑: Increased levels, ↓: Reduced levels, +: Reduced PHEC-66 cytotoxicity, −: Did not reduce PHEC-66 cytotoxicity, =: No change.

## Data Availability

The data for this manuscript is confidential. However, it can be provided to researcher if the request is reasonable.

## References

[1] Liu J., Fukunaga-Kalabis M., Li L., Herlyn M. (2014). Developmental pathways activated in melanocytes and melanoma. Arch. Biochem. Biophys..

[2] Saginala K., Barsouk A., Aluru J.S., Rawla P., Barsouk A. (2021). Epidemiology of Melanoma. Med. Sci..

[3] Zbytek B., Carlson J.A., Granese J., Ross J., Mihm M.C., Slominski A. (2008). Current concepts of metastasis in melanoma. Expert Rev. Dermatol..

[4] Huang Y.Y., Vecchio D., Avci P., Yin R., Garcia-Diaz M., Hamblin M.R. (2013). Melanoma resistance to photodynamic therapy: New insights. Biol. Chem..

[5] Volkovova K., Bilanicova D., Bartonova A., Letašiová S., Dusinska M. (2012). Associations between environmental factors and incidence of cutaneous melanoma. Review. Environ. Health.

[6] Nguyen D., Holien J., Dekiwadia C., Thrimawithana T., Piva T., Huynh T. (2023). *Momordica cochinchinensis* (Gấc) Seed Extracts Induce Apoptosis and Necrosis in Melanoma Cells. Pharmaceuticals.

[7] Sanchez J.N., Wang T., Cohen M.S. (2018). BRAF and MEK Inhibitors: Use and Resistance in BRAF-Mutated Cancers. Drugs.

[8] Chan X.Y., Singh A., Osman N., Piva T.J. (2017). Role Played by Signalling Pathways in Overcoming BRAF Inhibitor Resistance in Melanoma. Int. J. Mol. Sci..

[9] Eroglu Z., Ribas A. (2016). Combination therapy with BRAF and MEK inhibitors for melanoma: Latest evidence and place in therapy. Ther. Adv. Med. Oncol..

[10] Schulz A., Raetz J., Karitzky P.C., Dinter L., Tietze J.K., Kolbe I., Käubler T., Renner B., Beissert S., Meier F. (2022). Head-to-Head Comparison of BRAF/MEK Inhibitor Combinations Proposes Superiority of Encorafenib Plus Trametinib in Melanoma. Cancers.

[11] Davis J., Wayman M. (2022). Encorafenib and Binimetinib Combination Therapy in Metastatic Melanoma. J. Adv. Pract. Oncol..

[12] Carvajal R.D., Piperno-Neumann S., Kapiteijn E., Chapman P.B., Frank S., Joshua A.M., Piulats J.M., Wolter P., Cocquyt V., Chmielowski B. (2018). Selumetinib in Combination with Dacarbazine in Patients with Metastatic Uveal Melanoma: A Phase III, Multicenter, Randomized Trial (SUMIT). J. Clin. Oncol..

[13] Mercogliano M.F., Bruni S., Mauro F., Elizalde P.V., Schillaci R. (2021). Harnessing Tumor Necrosis Factor Alpha to Achieve Effective Cancer Immunotherapy. Cancers.

[14] Jiang T., Zhou C., Ren S. (2016). Role of IL-2 in cancer immunotherapy. OncoImmunology.

[15] Faghfuri E., Faramarzi M.A., Nikfar S., Abdollahi M. (2015). Nivolumab and pembrolizumab as immune-modulating monoclonal antibodies targeting the PD-1 receptor to treat melanoma. Expert Rev. Anticancer Ther..

[16] de Azevedo S.J., de Melo A.C., Roberts L., Caro I., Xue C., Wainstein A. (2021). First-line atezolizumab monotherapy in patients with advanced BRAF(V600) wild-type melanoma. Pigment Cell Melanoma Res..

[17] Rausch M.P., Hastings K.T., Ward W.H., Farma J.M. (2017). Immune Checkpoint Inhibitors in the Treatment of Melanoma: From Basic Science to Clinical Application. Cutaneous Melanoma: Etiology and Therapy.

[18] Moniuszko M., Radziwon P., Tucker S.C., Honn K.V. (2021). Inhibitors of immune checkpoints-PD-1, PD-L1, CTLA-4-new opportunities for cancer patients and a new challenge for internists and general practitioners. Cancer Metastasis Rev..

[19] Carlino M.S., Larkin J., Long G.V. (2021). Immune checkpoint inhibitors in melanoma. Lancet.

[20] Bachari A., Nassar N., Schanknecht E., Telukutla S., Piva T.J., Mantri N. (2024). Rationalizing a prospective coupling effect of cannabinoids with the current pharmacotherapy for melanoma treatment. Wiley Interdiscip. Rev. Syst. Biol. Med..

[21] Saad M.B., Hong L., Aminu M., I Vokes N., Chen P., Salehjahromi M., Qin K., Sujit S.J., Lu X., Young E. (2023). Predicting benefit from immune checkpoint inhibitors in patients with non-small-cell lung cancer by CT-based ensemble deep learning: A retrospective study. Lancet Digit. Health.

[22] Darvin P., Toor S.M., Sasidharan Nair V., Elkord E. (2018). Immune checkpoint inhibitors: Recent progress and potential biomarkers. Exp. Mol. Med..

[23] Smalley K.S., Eroglu Z., Sondak V.K. (2016). Combination Therapies for Melanoma: A New Standard of Care?. Am. J. Clin. Dermatol..

[24] Chinembiri T.N., Du Plessis L.H., Gerber M., Hamman J.H., Du Plessis J. (2014). Review of natural compounds for potential skin cancer treatment. Molecules.

[25] Bachari A., Piva T.J., Salami S.A., Jamshidi N., Mantri N. (2020). Roles of Cannabinoids in Melanoma: Evidence from In Vivo Studies. Int. J. Mol. Sci..

[26] Lu H.-C., Mackie K. (2016). An Introduction to the Endogenous Cannabinoid System. Biol. Psychiatry.

[27] Zou S., Kumar U. (2018). Cannabinoid Receptors and the Endocannabinoid System: Signaling and Function in the Central Nervous System. Int. J. Mol. Sci..

[28] Hermanson D.J., Marnett L.J. (2011). Cannabinoids, endocannabinoids, and cancer. Cancer Metastasis Rev..

[29] Ramer R., Schwarz R., Hinz B. (2019). Modulation of the Endocannabinoid System as a Potential Anticancer Strategy. Front. Pharmacol..

[30] Muller C., Morales P., Reggio P.H. (2018). Cannabinoid Ligands Targeting TRP Channels. Front. Mol. Neurosci..

[31] Preet A., Qamri Z., Nasser M.W., Prasad A., Shilo K., Zou X., Groopman J.E., Ganju R.K. (2011). Cannabinoid receptors, CB1 and CB2, as novel targets for inhibition of non-small cell lung cancer growth and metastasis. Cancer Prev. Res..

[32] Sarfaraz S., Afaq F., Adhami V.M., Mukhtar H. (2005). Cannabinoid receptor as a novel target for the treatment of prostate cancer. Cancer Res..

[33] Bachari A., Nassar N., Telukutla S., Zomer R., Dekiwadia C., Piva T.J., Mantri N. (2023). In Vitro Antiproliferative Effect of Cannabis Extract PHEC-66 on Melanoma Cell Lines. Cells.

[34] Dariš B., Tancer Verboten M., Knez Z., Ferk P. (2019). Cannabinoids in cancer treatment: Therapeutic potential and legislation. Bosn. J. Basic Med. Sci..

[35] Heider C.G., Itenberg S.A., Rao J., Ma H., Wu X. (2022). Mechanisms of Cannabidiol (CBD) in Cancer Treatment: A Review. Biology.

[36] Ramer R., Rohde A., Merkord J., Rohde H., Hinz B. (2010). Decrease of plasminogen activator inhibitor-1 may contribute to the anti-invasive action of cannabidiol on human lung cancer cells. Pharm. Res..

[37] Chen Y., Gibson S.B. (2008). Is mitochondrial generation of reactive oxygen species a trigger for autophagy?. Autophagy.

[38] Anis O., Vinayaka A.C., Shalev N., Namdar D., Nadarajan S., Anil S.M., Cohen O., Belausov E., Ramon J., Gati E.M. (2021). Cannabis-Derived Compounds Cannabichromene and Δ9-Tetrahydrocannabinol Interact and Exhibit Cytotoxic Activity against Urothelial Cell Carcinoma Correlated with Inhibition of Cell Migration and Cytoskeleton Organization. Molecules.

[39] Soliman E., Van Dross R. (2016). Anandamide-induced endoplasmic reticulum stress and apoptosis are mediated by oxidative stress in non-melanoma skin cancer: Receptor-independent endocannabinoid signaling. Mol. Carcinog..

[40] Pagano C., Savarese B., Coppola L., Navarra G., Avilia G., Laezza C., Bifulco M. (2023). Cannabinoids in the Modulation of Oxidative Signaling. Int. J. Mol. Sci..

[41] Donadelli M., Dando I., Zaniboni T., Costanzo C., Pozza E.D., Scupoli M.T., Scarpa A., Zappavigna S., Marra M., Abbruzzese A. (2011). Gemcitabine/cannabinoid combination triggers autophagy in pancreatic cancer cells through a ROS-mediated mechanism. Cell Death Dis..

[42] Massi P., Vaccani A., Bianchessi S., Costa B., Macchi P., Parolaro D. (2006). The non-psychoactive cannabidiol triggers caspase activation and oxidative stress in human glioma cells. Cell. Mol. Life Sci..

[43] McKallip R.J., Jia W., Schlomer J., Warren J.W., Nagarkatti P.S., Nagarkatti M. (2006). Cannabidiol-induced apoptosis in human leukemia cells: A novel role of cannabidiol in the regulation of p22*^phox^* and Nox4 expression. Mol. Pharmacol..

[44] Zhao R.-Z., Jiang S., Zhang L., Yu Z.-B. (2019). Mitochondrial electron transport chain, ROS generation and uncoupling (Review). Int. J. Mol. Med..

[45] Park S.-W., Hah J.H., Oh S.-M., Jeong W.-J., Sung M.-W. (2016). 5-lipoxygenase mediates docosahexaenoyl ethanolamide and N-arachidonoyl-L-alanine-induced reactive oxygen species production and inhibition of proliferation of head and neck squamous cell carcinoma cells. BMC Cancer.

[46] Vilema-Enríquez G., Arroyo A., Grijalva M., Amador-Zafra R.I., Camacho J. (2016). Molecular and Cellular Effects of Hydrogen Peroxide on Human Lung Cancer Cells: Potential Therapeutic Implications. Oxidative Med. Cell. Longev..

[47] DeMorrow S., Francis H., Gaudio E., Ueno Y., Venter J., Onori P., Franchitto A., Vaculin B., Vaculin S., Alpini G. (2008). Anandamide inhibits cholangiocyte hyperplastic proliferation via activation of thioredoxin 1/redox factor 1 and AP-1 activation. Am. J. Physiol. Gastrointest. Liver Physiol..

[48] Murad H., Hawat M., Ekhtiar A., AlJapawe A., Abbas A., Darwish H., Sbenati O., Ghannam A. (2016). Induction of G1-phase cell cycle arrest and apoptosis pathway in MDA-MB-231 human breast cancer cells by sulfated polysaccharide extracted from Laurencia papillosa. Cancer Cell Int..

[49] Guo M., Lu B., Gan J., Wang S., Jiang X., Li H. (2021). Apoptosis detection: A purpose-dependent approach selection. Cell Cycle.

[50] Limas J.C., Cook J.G. (2019). Preparation for DNA replication: The key to a successful S phase. FEBS Lett..

[51] McKenna S., García-Gutiérrez L., Matallanas D., Fey D. (2021). BAX and SMAC regulate bistable properties of the apoptotic caspase system. Sci. Rep..

[52] Peña-Blanco A., García-Sáez A.J. (2018). Bax, Bak and beyond-mitochondrial performance in apoptosis. FEBS J..

[53] Tsujimoto Y. (1998). Role of Bcl-2 family proteins in apoptosis: Apoptosomes or mitochondria?. Genes Cells.

[54] Park H.A., Broman K., Jonas E.A. (2021). Oxidative stress battles neuronal Bcl-xL in a fight to the death. Neural Regen. Res..

[55] Qian S., Wei Z., Yang W., Huang J., Yang Y., Wang J. (2022). The role of BCL-2 family proteins in regulating apoptosis and cancer therapy. Front. Oncol..

